# Supplementary material for: Genome-Wide Identification of GSTs Gene Family and Functional Analysis of BraGSTF2 of Winter Rapeseed (Brassica rapa L.) under Cold Stress
Source: Genes (Basel). 2023 Aug 25;14(9):1689. doi: 10.3390/genes14091689 (PMC10531308; doi:10.3390/genes14091689)
Supplement: Supplementary file 1 [file genes-14-01689-s001.zip › Supplementary Figure S1.pdf]

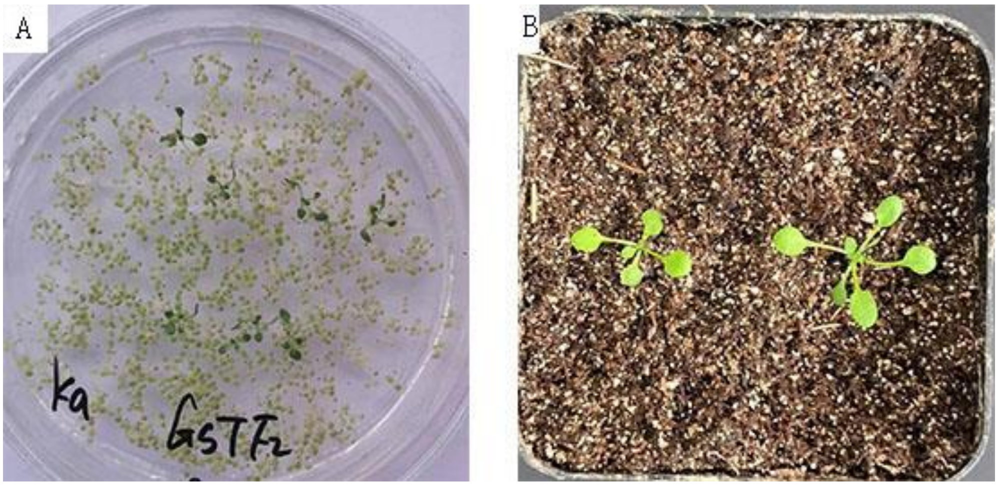

Supplementary Figure S1 Resistance screening of positive transgenic Arabidopsis thaliana

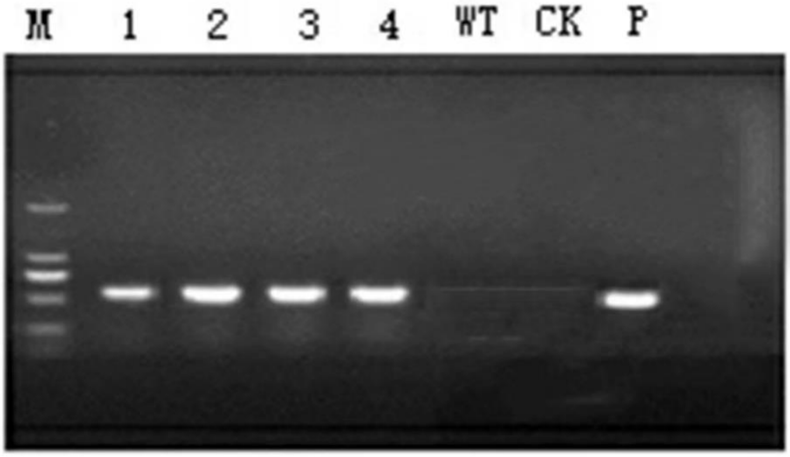

Supplementary Figure S2 PCR identification of positive transgenic plants of *BraGSTF2* overexpression

Note: M:DL2000 DNA Marker; swimming lane 1-4:T1 generation, a total of 4 positive strains; swimming lane WT: wild type plants; CK: water; P: positive control (Agrobacterium tumefaciens liquid).

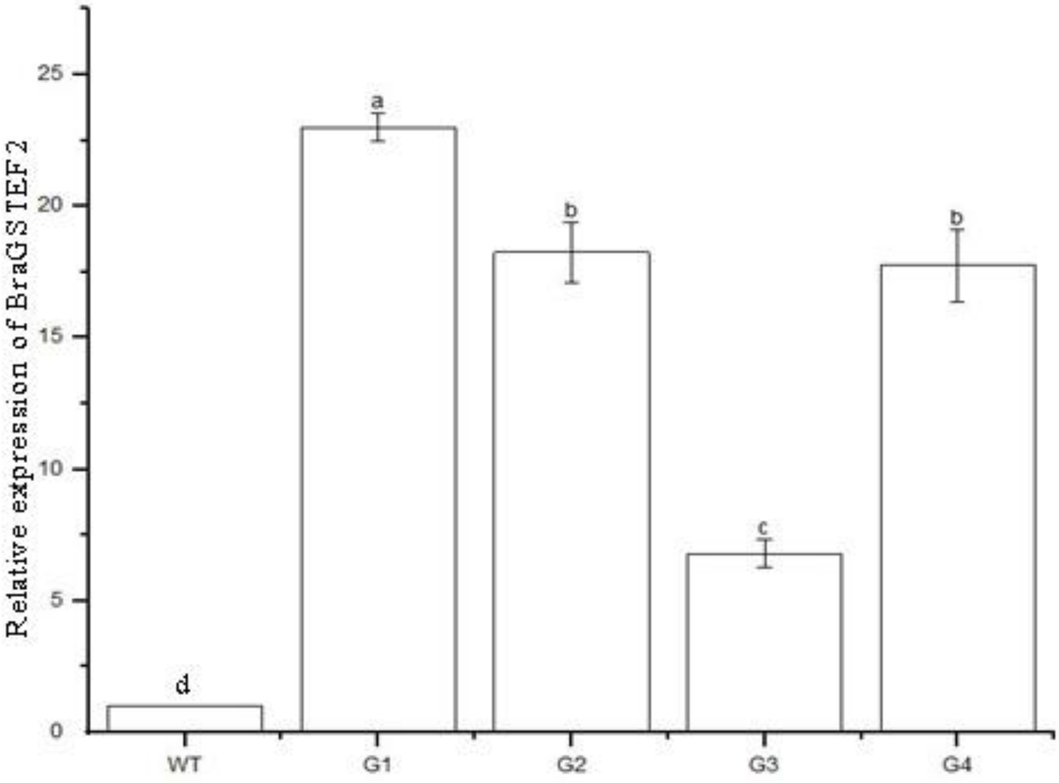

Supplementary Figure S3 RT-PCR test of transgenic Arabidopsis

Note: Different letters indicate the significance among different individual plants ($P < 0.05$). The error bar represents the standard error of the average of the sample. WT: wild type, G: transgenic Arabidopsis thaliana.
