# Supplementary material for: Genome-Wide Identification of GSTs Gene Family and Functional Analysis of BraGSTF2 of Winter Rapeseed (Brassica rapa L.) under Cold Stress
Source: Genes (Basel). 2023 Aug 25;14(9):1689. doi: 10.3390/genes14091689 (PMC10531308; doi:10.3390/genes14091689)
Supplement: Supplementary file 1 [file genes-14-01689-s001.zip › Supplementary Table S1.pdf]

Table S1 Prediction of physicochemical properties and secondary structure of *BraGST*

Protein name	Number of amino acids	pI	Instability index	Aliphatic index	Grand average of hydropathicity	Alpha helix	Beta bridge	Random coil
BraDHAR3	257	9.35	30.7	70.1	-0.4	15	4	47
BraDHAR1	210	6.83	36.8	96.1	0	30	11	86
BraDHAR4	213	5.79	42.1	103	-0.1	33	15	65
BraGSTF1	213	5.66	38.1	90.2	-0.3	33	14	72
BraGSTF10	215	6.76	28.1	90.7	-0.2	33	13	71
BraGSTF11	210	6.41	28	93	-0.3	35	12	70
BraGSTF12	213	5.13	39	87.8	-0.2	19	7	61
BraGSTF13	215	5.76	43.5	90.6	-0.2	33	9	85
BraGSTF14	254	6.67	34.3	93	-0.3	34	12	71
BraGSTF15	248	6.25	35.5	89.3	-0.4	25	13	62
BraGSTF16	210	7.71	33	91.5	-0.2	33	14	81
BraGSTF17	231	5.98	38.6	90.2	-0.2	31	11	75
BraGSTF18	264	5.79	35.9	96.6	-0.1	15	10	47
BraGSTF19	215	6.17	38.7	90.2	-0.2	34	16	70
BraGSTF2	213	5.14	36.7	97.8	-0.1	30	18	62
BraGSTF20	215	5.91	35.3	84.4	-0.3	32	9	65
BraGSTF3	213	5.05	40.6	89.4	-0.3	20	10	77
BraGSTF4	213	5.98	39.2	88.6	-0.3	23	11	77
BraGSTF5	213	5.44	39.2	83.6	-0.3	31	8	67
BraGSTF6	251	5.1	28.5	79.7	-0.3	34	12	64
BraGSTF7	215	5.43	38	82.8	-0.4	31	12	61
BraGSTF8	181	5.11	38.3	87.7	-0.3	29	17	55
BraGSTF9	482	5.45	44.5	80.1	-0.3	31	12	58
BraGSTL1	297	5.32	36.9	83.7	-0.3	25	10	63
BraGSTL2	459	6.36	37.7	77.4	-0.5	29	14	67
BraGSTT1	241	5.29	32.1	81.1	-0.4	36	9	64
BraGSTT2	246	5.75	32	93.5	-0.2	29	7	55
BraGSTT3	181	5.42	37.7	87.7	-0.3	26	14	5
BraTCHQD	266	5.33	33.8	79.2	-0.4	36	10	67
BraGSTU1	232	6.13	37.6	100	-0.3	26	12	63
BraGSTU10	227	6.04	47.7	95.3	-0.3	30	12	58
BraGSTU11	224	6.17	57.4	95.3	-0.2	28	11	57
BraGSTU12	249	6.04	44.2	98.4	-0.3	29	11	62
BraGSTU13	227	7.71	34.7	95.7	-0.2	26	11	60
BraGSTU14	227	5.17	41.9	106	-0.1	28	10	60
BraGSTU15	219	5.28	43.4	86.7	-0.3	28	7	53
BraGSTU17	224	6.14	34.7	87.3	-0.1	26	9	67
BraGSTU18	116	5.84	43	90.5	-0.4	34	7	72
BraGSTU19	221	5.61	34.8	94.1	-0.2	25	9	64
BraGSTU2	232	6.13	36.9	91.2	0	26	13	59

BraGSTU20	164	5.59	47.7	100	-0.1	25	9	63
BraGSTU21	218	9.17	53.6	78.3	-0.3	30	11	62
BraGSTU22	220	5.49	36.1	91.1	0	27	12	64
BraGSTU23	215	4.84	31.2	85.1	-0.1	28	13	59
BraGSTU24	219	5.32	44.2	92.8	-0.1	30	11	64
BraGSTU25	219	7.71	30.6	99.7	-0.1	30	16	7
BraGSTU27	219	5.47	43.6	99.1	-0.3	29	17	56
BraGSTU28	219	5.86	33.4	101.6	-0.3	28	12	58
BraGSTU29	219	5.57	56.7	101.5	-0.3	27	16	60
BraGSTU3	234	5.23	44.9	91.6	-0.1	35	12	68
BraGSTU30	220	9.5	44.9	93.5	-0.2	21	15	88
BraGSTU31	227	9.36	42.1	92.8	-0.2	29	14	82
BraGSTU32	224	9.11	52	81.1	-0.3	32	14	75
BraGSTU33	228	5.31	36.4	97.1	-0.2	25	11	67
BraGSTU34	398	9.12	27.1	88.9	-0.4	30	16	77
BraGSTU35	224	5.16	40	85.6	-0.3	27	20	81
BraGSTU36	224	6.98	32.4	83.1	-0.2	30	14	73
BraGSTU37	224	8.32	35.7	80.8	-0.3	38	6	92
BraGSTU38	225	8.51	48.4	89.1	-0.1	42	22	94
BraGSTU39	226	9.28	47.6	92.2	-0.4	30	12	61
BraGSTU4	182	6.84	51.7	82.8	-0.4	37	7	152
BraGSTU26	234	6.7	39	71	-0.5	49	8	162
BraGSTU5	230	5.06	43	86	-0.2	64	46	133
BraGSTU6	255	5.58	31.9	79.4	-0.3	60	11	175
BraGSTU7	228	5.54	33.7	77.2	-0.3	62	12	164
BraGSTU8	228	4.99	35.4	87.9	-0.3	50	19	146
BraGSTU9	228	9.04	50.4	88.6	-0.1	84	32	205
BraGSTZ1	216	5.39	39.8	100.8	0.2	147	44	208
BraGSTZ2	600	9.54	43.8	78.8	-0.4	109	23	290
BraGSTU16	114	5.52	54.2	71.8	-0.5	18	6	27
